# The Application of Liver Stiffness Measurement in Residents Without Overt Liver Diseases Through a Community-Based Screening Program: Erratum

**DOI:** 10.1097/01.md.0000484785.53640.44

**Published:** 2016-06-17

In the article “The Application of Liver Stiffness Measurement in Residents Without Overt Liver Diseases Through a Community-Based Screening Program”,^1^ which appeared in Volume 95, Issue 12 of *Medicine*, errors were detected in Table 2. The article has since been corrected online.
